# Synergistic activity of caspofungin and posaconazole against *Candida* (*Candidozyma*) *auris* biofilms based on phenotypic, transcriptomic, and *in vivo* insights

**DOI:** 10.1128/spectrum.02686-25

**Published:** 2026-03-12

**Authors:** Fruzsina Kovács, Andrea Harmath, Dávid Balázsi, Lajos Forgács, Ágota Ragyák, Noémi Balla, Zoltán Tóth, Aliz Bozó, Andrew M. Borman, László Majoros, Renátó Kovács, Ágnes Jakab

**Affiliations:** 1 Department of Medical Microbiology, Faculty of Medicine, University of Debrecen, Debrecen, Hungary; 2 Doctoral School of Pharmaceutical Sciences, University of Debrecen 37599, Debrecen, Hungary; 3 Department of Inorganic and Analytical Chemistry, Agilent Atomic Spectroscopy Partner Laboratory, University of Debrecen 37599, Debrecen, Hungary; 4 Medical Microbiology, Clinical Center, University of Debrecen, Debrecen, Hungary; 5 UK National Mycology Reference Laboratory, UK Health Security Agency, Science Quarter, Southmead Hospital 436286 , Bristol, United Kingdom; 6 Medical Research Council Centre for Medical Mycology (MRC CMM), University of Exeter 3286, Exeter, United Kingdom; Mycology Laboratory, Wadsworth Center, Albany, New York, USA

**Keywords:** *Candida auris*, transcriptome, posaconazole, caspofungin, echinocandin, antifungal therapy, ergosterol, synergy, mouse, *in vivo*

## Abstract

*Candida* (*Candidozyma*) *auris* is an emerging multidrug-resistant fungal pathogen capable of establishing persistent skin colonization, contaminating the environment, and causing nosocomial outbreaks associated with high mortality rates. Conventional monotherapy frequently proves inadequate against biofilm-associated infections, underscoring the urgent need for novel therapeutic strategies. Therefore, we investigated the physiological and molecular responses of South Asian clade *C. auris* biofilms to treatment with a caspofungin–posaconazole combination. This regimen markedly reduced the median minimum inhibitory concentrations (4- to 32-fold for caspofungin; 8- to 64-fold for posaconazole) compared with monotherapies. Synergistic interactions were observed in all isolates, with fractional inhibitory concentration indices ranging from 0.078 to 0.31, and were further confirmed *in vivo*. Transcriptomic profiling revealed activation of multiple stress-response pathways, driving adaptive changes such as enhanced extracellular matrix production and biofilm-forming capacity, maintenance of intracellular cation homeostasis, osmotic stress response, and extensive cell wall and membrane remodeling affecting the mannan–glucan complex, chitin, sphingolipids, phosphatidylinositol-(4,5)-bisphosphate, and ergosterol content. Additional responses included activation of *RCT1* (fluconazole-inducible protein) and *MDR1* (drug efflux pump), collectively promoting survival under combined antifungal pressure. These findings demonstrate the potent synergistic activity of caspofungin and posaconazole against *C. auris* biofilms, thereby supporting the development of effective combination therapies for this high-risk pathogen.

**IMPORTANCE:**

*Candida auris* is a rapidly emerging fungal pathogen that presents substantial challenges for infection control owing to its multidrug resistance, persistence in healthcare environments, and capacity to cause large-scale outbreaks. Biofilm formation on indwelling medical devices plays a pivotal role in *C. auris* outbreaks within healthcare settings and is implicated in nearly 90% of *C. auris* candidemia cases. These biofilms also exhibit pronounced tolerance to antifungal agents, thereby restricting available treatment options. Our study demonstrates that the combination of caspofungin and posaconazole exerts a strong synergistic effect against *C. auris* biofilms, both *in vitro* and *in vivo*. By elucidating the molecular mechanisms behind this synergy—including stress-response activation, cell wall and membrane remodeling, calcium signaling, and regulation of drug efflux pumps—this work provides important insights into antifungal therapeutic responses in *C. auris* and underscores combination therapy as a promising strategy to overcome biofilm-associated antifungal resistance in this high-risk pathogen.

## INTRODUCTION

The World Health Organization (WHO) has designated *Candida* (*Candidozyma*) *auris* as a critical public health threat due to its multidrug resistance to antifungal agents, high mortality rate (30%–72%), and its ability to cause nosocomial outbreaks, particularly among hospitalized and immunocompromised patients (1–4). While azole antifungals are commonly used as first-line agents against systemic *Candida* infections, echinocandins are the treatment of choice for systemic *C. auris* infections due to its resistance to fluconazole, observed in up to 90% of isolates (5–7). Alarmingly, over 40% of *C. auris* isolates have been reported to be multidrug resistant, and approximately 8% exhibit pan-resistance to azoles, polyenes, and echinocandins, particularly among South Asian clade isolates (4–6, 8).

*C. auris* frequently colonizes indwelling medical devices, such as central venous catheters, mechanical ventilators, and urinary catheters, as well as various hospital surfaces. This promotes nosocomial transmission and facilitates biofilm formation, which significantly increases resistance to antifungal agents (9–13). Given the limited therapeutic options for treating *C. auris* biofilm-associated infections, drug repurposing and the use of drug combinations represent promising alternative approaches. These strategies can enhance antifungal efficacy through synergistic mechanisms of action, reduce toxicity, delay the emergence of resistance, and ultimately improve patient outcomes (9, 14, 15). Recently, *in vitro* and *in vivo* studies have demonstrated that the combination of caspofungin and posaconazole exhibits broad-spectrum activity against *Aspergillus fumigatus* (16), *A. flavus* (17), *Zygomycetes* (18), *C. albicans, C. krusei*, and *C. glabrata* (19–22). Furthermore, synergistic effects have been reported against planktonic (23) and echinocandin-resistant *C. auris* isolates (24). However, the efficacy of this combination against *C. auris* biofilms—particularly regarding global transcriptional responses and *in vivo* effectiveness—remains insufficiently explored. Therefore, the present study aims to assess the *in vitro* and *in vivo* antifungal activity of posaconazole in combination with caspofungin against sessile *C. auris* isolates belonging to the South Asian clade. Our findings suggest a novel alternative therapeutic approach for treating *C. auris* biofilm-associated infections, thereby contributing to the advancement of more effective antifungal strategies.

## MATERIALS AND METHODS

### *Candida auris* isolates

Six isolates of South Asian *C. auris* clade were obtained from the National Mycology Reference Laboratory (United Kingdom; Table S1). Species-level identification was performed using matrix-assisted laser desorption/ionization time-of-flight mass spectrometry (MALDI-TOF MS) with Microflex Biotyper (Bruker Daltonics, Bremen, Germany) using the manufacturer’s standard protocols. For the biofilm viability assays, RNA sequencing-based transcriptomic analyses, *in vivo* experiments, and elemental analysis of biofilms, isolate 10 (*C. auris* NCPF 8971) was selected and used as a representative isolate.

### Biofilm development assay

All *C. auris* strains were cultured in yeast extract–peptone–dextrose (YPD) agar plates (1% yeast extract [Alfa Aesar, USA], 2% mycological peptone [Oxoid, United Kingdom], 2% dextrose, and 2% agar [VWR International LLC, Hungary], pH 5.6) for 48 h at 37°C. Cell suspensions were prepared in RPMI-1640 medium (with L-glutamine and without bicarbonate, pH 7.0, and with MOPS; Merck, Hungary) at a concentration of 1 × 10⁶ CFU/mL using a Bürker chamber. Then, 100 µL aliquots were inoculated into 96-well flat-bottom microtiter plates (TPP, Switzerland). After incubation at 37°C for 24 h, non-adherent cells were removed by washing with sterile physiological saline. Biofilm formation was evaluated using two methods: (i) the crystal violet (CV) assay to measure total biomass and (ii) the XTT (2,3-bis-[2-methoxy-4-nitro-5-sulfophenyl]−2H-tetrazolium-5-carboxanilide; Merck, Hungary) reduction assay to assess metabolic activity, as described previously (24–29). Biofilm-forming capacity was classified using quartile-based thresholds established from CV assay-derived biomass measurements. Of the 21 South Asian clade isolates available in our laboratory, those selected for the present study exhibited the highest biofilm biomass (the optical density [OD] at 540 nm ranged from 0.118 to 0.914). Isolates in the first quartile were classified as non-biofilm producers, those within the interquartile range as biofilm producers, and isolates with biomass values above the third quartile as strong biofilm producers. Wells containing culture medium only were used as negative controls to account for background crystal violet staining, ensuring that the measured absorbance values reflected true biofilm-associated biomass.

### Assessment of antifungal susceptibility for planktonic cells and biofilms

To determine the minimum inhibitory concentrations (MICs), drug concentration ranges were as follows: 0.008–2.0 mg/L for posaconazole (Merck, Hungary) and 0.5–32 mg/L for caspofungin (Merck, Hungary), respectively. The planktonic MIC was determined according to the recommendations proposed by the Clinical Laboratory Standards Institute M27-A3 protocol. *C. parapsilosis* ATCC 22 019 and *C. krusei* ATCC 6258 were used as quality control strains (30). Biofilms were incubated at 37°C for 0, 3, 6, 9, 12, or 24 h with the respective antifungal agents. After incubation, the wells were washed with sterile physiological saline, and biofilm viability was assessed using the XTT assay as described previously (26, 29). The percentage change in metabolic activity was calculated based on absorbance at 492 nm using a microplate spectrophotometer, according to the following formula: 100% × (OD_well_ − OD_background_)/(OD_drug-free well_ − OD_background_). The OD_background_ corresponds to 100 µL of XTT solution without biofilm and without antifungal agent. The MICs were defined as the lowest drug concentration resulting in at least a 50% reduction in metabolic activity compared with the untreated control. MIC values are reported as the median of three independent experiments performed per isolate.

### Assessment of synergy between caspofungin and posaconazole

The drug–drug interactions between caspofungin and posaconazole were assessed using a two-dimensional checkerboard broth microdilution assay, as previously described (24–26). The interactions were evaluated by calculating the fractional inhibitory concentration index (FICI), defined as ΣFIC = FIC_A_ + FIC_B_ = [(MIC_A_^comb^/MIC_A_^alone^)] + [(MIC_B_^comb^/MIC_B_^alone^)], where MIC_A_^alone^ and MIC_B_^alone^ represent the MICs of caspofungin and posaconazole when used alone, and MIC_A_^comb^ and MIC_B_^comb^ represent the MICs of drugs in combination at isoeffective combinations. The FICI values were determined as the lowest ΣFIC. MICs, both alone and in combination, were defined as the lowest concentrations that resulted in at least a 50% reduction in metabolic activity compared with the untreated control biofilms. Off-scale MIC values were converted to the next highest twofold concentration. Synergism was defined as FICI ≤ 0.5, an indifferent interaction as FICI between >0.5 and 4, and antagonism as FICI > 4. FICI values were determined in three independent experiments and are presented as the median value.

### Biofilm viability assay

One-day-old biofilms were developed on the surface of a four-well Permanox plastic slide (Lab-Tek Chamber Slide System, VWR, Hungary). Preformed biofilms were washed three times with sterile physiological saline. Caspofungin (1 mg/L) and posaconazole (0.25 mg/L), either alone or in combination, were added and incubated at 37°C for 24 h. Drug concentrations were selected based on the results of the checkerboard assay. Following treatment, biofilms were washed with sterile physiological saline, and the ratio of viable to dead cells was determined using the fluorescent LIVE/DEAD BacLight viability kit (Thermo Fisher Scientific, USA), as described in our previous work (24, 29). Live and non-viable cells were stained with Syto 9 and propidium iodide, respectively. Fluorescent cells were visualized with a Zeiss AxioSkop 2 mot microscope (Zeiss, Germany) equipped with a Zeiss AxioCam HRc camera (Zeiss, Germany). Image analysis was conducted with Axiovision 4.8.2 (Zeiss, Germany). The percentage of dead cells was calculated using ImageJ software (version: 2.9.0/1.53t; Fiji distribution, developed by Wayne Rasband, National Institutes of Health, USA). Data are presented as mean ± standard deviation (SD) from three independent experiments performed per isolate 10. Statistical analysis was performed using one-way analysis of variance (ANOVA), followed by Dunnett’s multiple comparisons test, with the untreated control serving as the reference group. This approach was chosen to specifically evaluate the effect of each treatment relative to the baseline condition. A two-sided *P* value < 0.05 was considered statistically significant. All analyses were conducted using standard parametric statistical methods.

### RNA-Seq and transcriptome analysis

One-day-old biofilms of isolate 10, treated with caspofungin (1 mg/L) and/or posaconazole (0.25 mg/L), were scraped from tissue culture dishes (TPP, Switzerland), pooled, and washed three times with physiological saline. Three biological replicates of the biofilm-forming cell suspensions were centrifuged at 3,000 × *g* for 10 min at 4°C. The resulting pellets were used for RNA extraction and intracellular metal content measurement (28).

Total RNA was extracted from lyophilized yeast cells derived from four different biofilms of isolate 10—untreated, caspofungin-treated, posaconazole-treated, and caspofungin with posaconazole-treated—using three biological replicates per condition. RNA yield and quality were assessed using a ND-1000 spectrophotometer (Nanodrop Technology, Thermo Fisher Scientific, USA) and a BioAnalyzer system (Agilent Technologies, USA), following the manufacturers’ protocols (28, 31). RNA library construction, sequencing, and data analysis were performed by the Genomic Medicine and Bioinformatics Core Facility, Department of Biochemistry and Molecular Biology, Faculty of Medicine, University of Debrecen, Debrecen, Hungary. For cDNA library preparation, the NEBNext RNA Sample Preparation Kit (New England BioLabs, USA) was used according to the manufacturer’s protocol. Sequencing was conducted on an Illumina NextSeq 2000 platform (Illumina, USA), generating single-end reads of 100 bp in length, with approximately 20.4 to 27.3 million reads per sample. Raw sequence quality was evaluated using the FastQC package (http://www.bioinformatics.babraham.ac.uk/projects/fastqc). Sequencing reads were aligned to the *C. auris* B8441 reference genome (*C. auris* genome version gca002759435.2; https://www.ncbi.nlm.nih.gov/datasets/genome/GCA_002759435.2) with the HISAT2 v2.1 alignment program in combination with SAMtools to generate read counts (32). Downstream analysis was performed using StrandNGS 4.0 software, and the integrated DESeq algorithm was applied to obtain normalized gene expression values. Principal component analysis (PCA) of the transcriptomes, based on normalized expression values, was performed using StrandNGS 4.0 software. Differentially expressed genes (DEGs) were identified using a moderated *t*-test with Benjamini-Hochberg false discovery rate (FDR) correction for multiple testing. Genes with a corrected *P*-value < 0.05 and a fold change (FC) > 1.5 or < −1.5 were considered upregulated or downregulated DEGs, respectively. Additionally, the enrichment analysis of the DEGs was performed using the *Candida* Genome Database Gene Ontology Term Finder (http://www.candidagenome.org/cgi-bin/GO/goTermFinder) and FungiDB (https://fungidb.org/fungidb/app/) to identify enriched biological processes, cellular components, and molecular functions. Figures were generated using GraphPad Prism software (version 10.0) and the SRPlot platform (http://www.bioinformatics.com.cn/SRplot).

### Validation of RNA-Seq results by RT-qPCR

RT-qPCR analysis was performed as previously described (28, 29, 31). Following the DNase treatment with DNase I (Merck, Hungary), the Luna Universal One-Step RT-qPCR Kit (New England Biolabs, USA) was used according to the manufacturer’s protocol, with oligonucleotide primer pairs listed in Table S1. The reaction mixtures contained the following components: 10 μL reaction mix, 1 μL enzyme mix, 10 μM of the forward and reverse primers, 5 μL RNA, and nuclease-free water to a final volume of 20 μL. All experiments were performed in triplicate, with three independent experiments for each sample. Relative gene expression values were calculated using ΔCP values, which represent the difference in crossing point (CP) cycle numbers between the reference and target genes within a sample. *C. auris* actin gene (B9J08_000486) was used as the reference gene (Table S1).

### Elemental analysis of biofilms using inductively coupled plasma optical emission spectrometry

Lyophilized biofilm samples of isolate 10 were weighed into glass beakers using an analytical balance (Precisa ES 225SM-DR) and subjected to atmospheric wet digestion. Each sample was treated with a mixture of 3 mL of 65% (m/m) nitric acid (HNO_3_; reagent grade, Merck, Hungary) and 1 mL of 30% (m/m) hydrogen peroxide (H_2_O_2_, reagent grade, Merck, Hungary). Following digestion, the samples were transferred into calibrated plastic centrifuge tubes and diluted to a final volume of 12.00 mL using 0.1 M HNO₃ prepared with ultrapure water (Synergy UV, Millipore). Samples were stored at room temperature prior to analysis.

The elemental composition was determined using inductively coupled plasma optical emission spectrometry (ICP-OES 5110 Vertical Dual View, Agilent Technologies, USA) equipped with an autosampler (Agilent SPS4), a Meinhard-type nebulizer, and a double pass spray chamber. Standard solutions for Ca, K, Mg, Na, P, and S were prepared from the mono element spectroscopic standards (1,000 mg/L; Scharlau), while standards for Ag, Al, B, Ba, Cd, Co, Cu, Cr, Fe, Li, Mn, Ni, Pb, Sr, and Zn were obtained from a multi-element spectroscopic standard solution (ICP IV, Merck, Hungary), also at a concentration of 1,000 mg/L. A five-point calibration curve was constructed for each element by diluting standards with 0.1 M HNO₃ in ultrapure water (28, 31). Mean ± SD values were calculated from three independent experiments. Statistical significance of changes was determined by a paired Student’s *t* test. The *P* value < 0.05 was considered statistically significant.

### Infection model

Immunocompromised female BALB/c mice (weighing 22–24 g) were used for the *in vivo* experiments. Immunosuppression was induced by intraperitoneal injection of cyclophosphamide (Endoxan, Baxter, Deerfield, IL, USA) at a dose of 150 mg/kg body weight 4 days before infection and 100 mg/kg 1 day before infection. Additional doses of 100 mg/kg were administered on days 2 and 4 post-infection. On day 0, mice were infected intravenously via the lateral tail vein with *C. auris* isolate 10 (1 × 10⁷ colony-forming units [CFU] in 200 μL of physiological saline). The actual inoculum density was verified by plating serial dilutions on Sabouraud dextrose agar.

Mice were assigned to four groups (8 mice per group): (i) untreated control; (ii) 1 mg/kg/day caspofungin; (iii) 1.5 mg/kg/day posaconazole; and (iv) combination of 1 mg/kg/day caspofungin and 1.5 mg/kg/day posaconazole. In the case of the caspofungin–posaconazole combination, posaconazole doses were administered 1 h after the caspofungin treatments. All therapies were given intraperitoneally and started 24 h post-infection (day 1). Control animals received 0.5 mL of sterile physiological saline intraperitoneally. On day 7, the animals were euthanized, and their kidneys were aseptically removed, weighed, and homogenized in sterile physiological saline. Homogenates were serially diluted and plated to determine CFU counts following incubation for 48 h at 37°C. Kidney fungal burden was analyzed using the Kruskal–Wallis test with Dunn’s *post hoc* test (GraphPad Prism 10.5.0). A *P*-value < 0.05 was considered statistically significant. Kidneys from both treated and untreated mice were subjected to histological analysis on day 7. Kidneys were fixed in formalin, embedded in paraffin, and sectioned at 4 µm. Tissue sections were stained with Grocott methenamine silver (25, 26, 33–35).

## RESULTS

### The physiological consequences of the caspofungin and posaconazole combination

All tested *C. auris* isolates were capable of forming biofilms, as confirmed by the CV assay. The median values and ranges of MICs for planktonic and sessile cells are shown in Table 1. In the case of planktonic cells, MICs ranged from 0.5 to >8 mg/L and from 0.015 to >0.125 mg/L for caspofungin and posaconazole, respectively. The median MICs observed in combination showed a 2- to 8-fold and a 2- to 75-fold reduction for caspofungin and posaconazole, respectively (Table 1). The results of the caspofungin with posaconazole interaction based on FICI are summarized in Table 1. Indifferent interaction was observed for 83% of the planktonic isolates (all median FICIs = 1).

**TABLE 1:** *In vitro* interaction between caspofungin and posaconazole against *Candida auris* planktonic cells and 1-day-old biofilms

Isolates	Median MIC (range) values^*a*^				Interaction analysis: FICI	
	Alone		In combination			
	Caspofungin (mg/L)	Posaconazole (mg/L)	Caspofungin (mg/L)	Posaconazole (mg/L)	Median (range) FICI	Interaction
Planktonic cells						
10	1	0.015 (0.015–0.06)	0.5	0.008 (0.008–0.03)	1	Indifferent
12	1	0.03	0.5 (0.5–1)	0.015	1 (1–1.5)	Indifferent
27	1	0.03 (0.015–0.03)	1	0.03 (0.0004–0.03)	1 (1–2)	Indifferent
82	>8^*b*^	0.06 (0.03–0.06)	1 (0.125–1)	0.0019	0.38 (0.19–0.38)	Synergy
88	0.5	0.03	0.5	0.0004	1	Indifferent
213	1	>0.125^*b*^	0.5	0.125	1	Indifferent
Biofilms						
10	>32^*c*^	1	8 (4–8)	0.125 (0.125–0.25)	0.14 (0.14–0.25)	Synergy
12	>32^*c*^	4	8 (4–8)	0.125 (0.06–0.125)	0.31 (0.25–0.375)	Synergy
27	>32^*c*^	2 (2–4)	1 (1–2)	0.25 (0.06–0.25)	0.14 (0.078–0.26)	Synergy
82	>32^*c*^	4	1 (1–2)	0.06 (0.06–0.125)	0.078 (0.048–0.078)	Synergy
88	16 (8–16)	0.5	1 (0.5–1)	0.03 (0.03–0.06)	0.25 (0.1875–0.37)	Synergy
213	>32^*c*^	1	2 (1–2)	0.125 (0.06–0.125)	0.28 (0.27–0.32)	Synergy

^*a*^ Fifty percent OD reduction in metabolic activity.

^*b*^ MIC is off-scale at >8 mg/L and >0.125 mg/L; 16 mg/L and 0.25 mg/L (one dilution higher than the highest tested concentration) were used for analysis.

^*c*^ MIC is off-scale at >32 mg/L; 64 mg/L (one dilution higher than the highest tested concentration) was used for analysis.

In contrast, *C. auris* biofilms showed significantly higher resistance to caspofungin and posaconazole compared with planktonic cells. The MICs of caspofungin and posaconazole alone ranged from 16 to >32 mg/L and from 0.5 to 4 mg/L, respectively. In combination treatment, the median MICs of caspofungin and posaconazole were reduced by 4- to 32-fold and 8- to 64-fold, respectively, compared to caspofungin or posaconazole alone (Table 1). Synergistic interactions were observed in all isolates tested, with FICIs ranging from 0.078 to 0.31 for sessile cells (Table 1).

**Fig 1:**
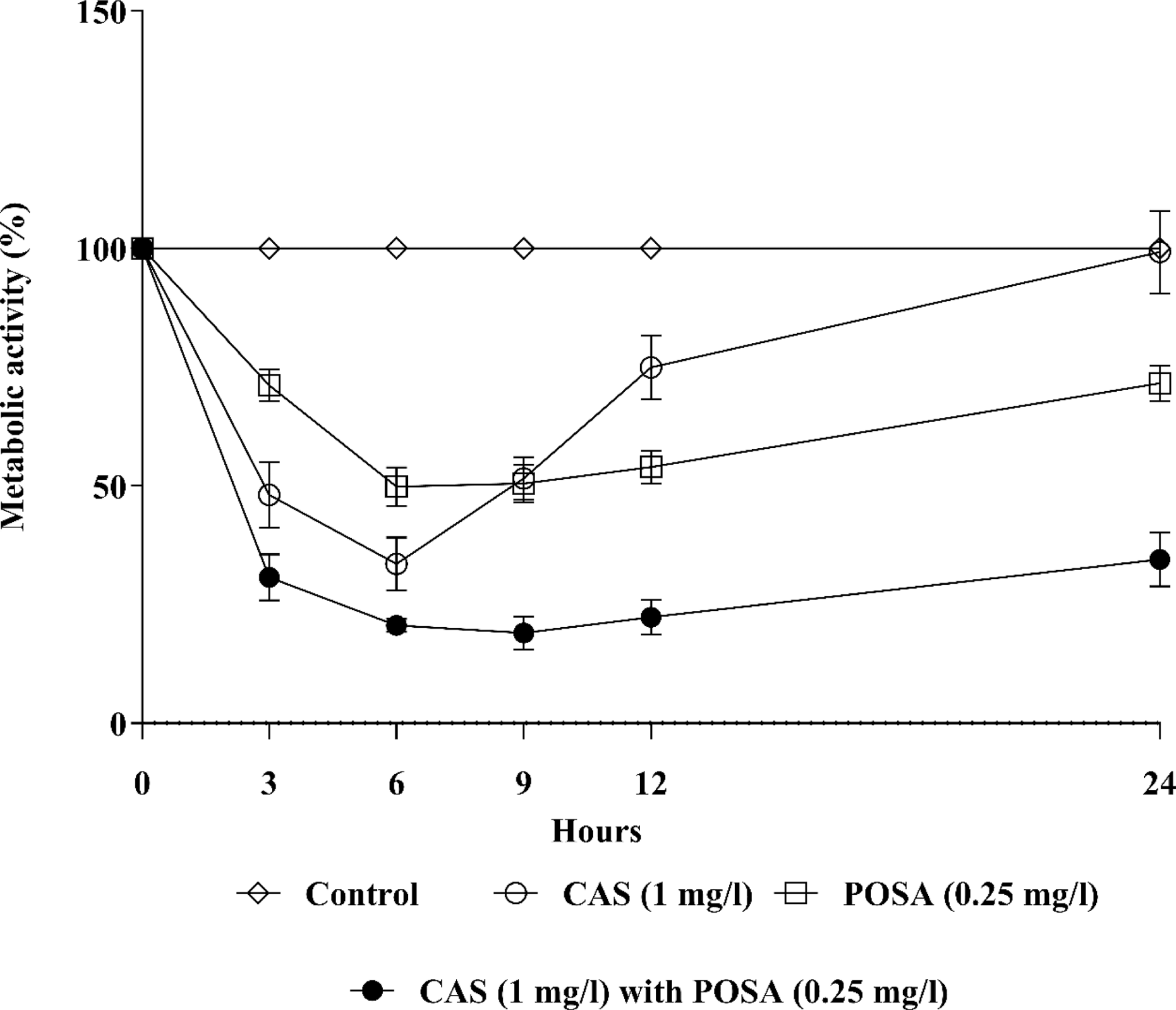
Metabolic activity changes over time in case of biofilm formation in the presence of caspofungin (CAS; 1 mg/L), posaconazole (POSA; 0.25 mg/L), and caspofungin (1 mg/L) with posaconazole (0.25 mg/L) for *Candida auris*. Each time point represents the mean ± SEM (standard error of mean) of metabolic activity of clinical isolates (three independent experiments per isolate).

In the case of sessile cells, the metabolic activity-based growth curves further confirmed the interactions observed in the FICI analysis (Fig. 1). For all isolates, a marked reduction in metabolic activity was observed at 6 h across all treatment groups (*P* < 0.001). Caspofungin alone significantly reduced the metabolic activity of biofilms between 3 h and 12 h compared to untreated controls (*P* < 0.01–0.001); however, metabolic activity returned to near control levels by 24 h. In contrast, posaconazole alone significantly reduced biofilm mass from 3 h to 24 h (*P* < 0.001) relative to controls. Notably, the combination of 1 mg/L caspofungin and 0.25 mg/L posaconazole resulted in a consistent and significant reduction in biofilm formation at all examined time points (*P* < 0.001) (Fig. 1).

**Fig 2:**
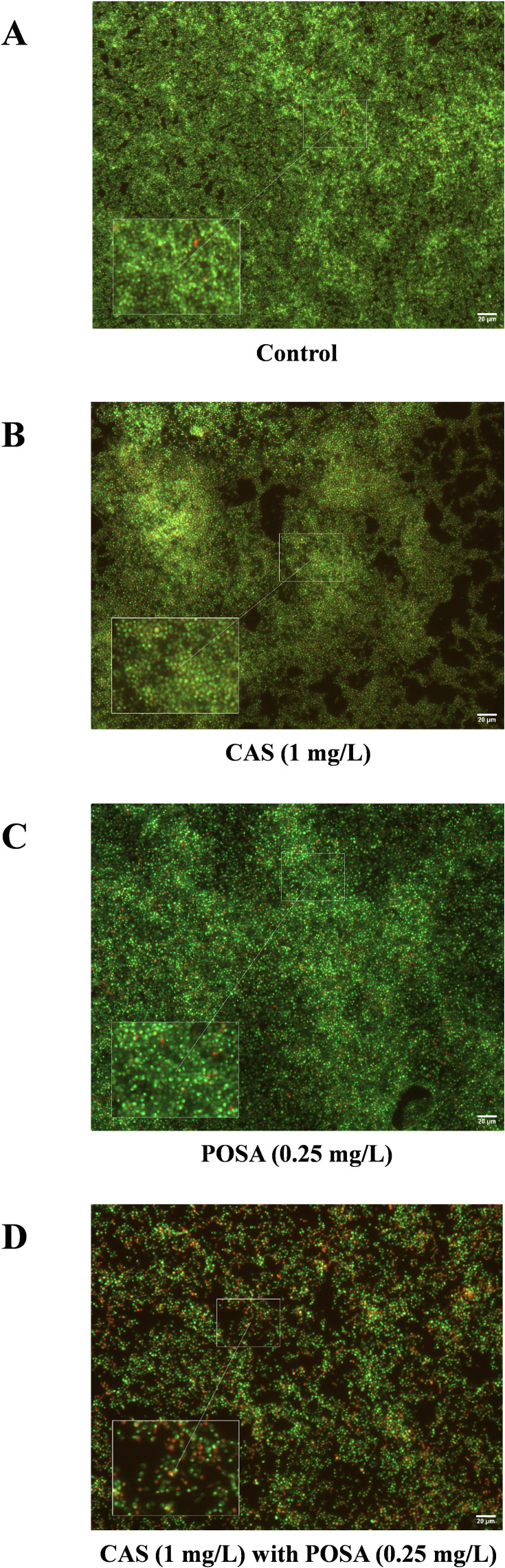
Yeast viability assay of *Candida auris* biofilm. LIVE/DEAD fluorescence imaging of one *C. auris* representative isolate (10) after caspofungin (**B**; CAS) or posaconazole (**C**; POSA) exposure. Picture **A** shows the untreated *C. auris* biofilm, while picture **D** demonstrates the caspofungin with posaconazole-exposed biofilm, respectively. Following 24 h of antifungal treatment, vital live cells (green) versus nonviable cells (red) were stained with Syto 9 and propidium iodide. All images show typical fields of view. Scale bars represent 20 µm.

Viability assays demonstrated that *C. auris* (isolate 10) biofilms treated with caspofungin (1 mg/L) exhibited increased cell death in the presence of posaconazole (0.25 mg/L) (Fig. 2D) compared to untreated control (Fig. 2A) or treatments with caspofungin or posaconazole alone (Fig. 2B and C). The proportion of dead cells was 12.5% ± 0.7% following caspofungin treatment, 29.9% ± 1.2% with posaconazole, and 52.6% ± 2.7% with the combination exposure compared to untreated control (0.46 ± 0.04%; *P* < 0.001).

### Genome-wide transcriptional change in mature *Candida auris* biofilms

**Fig 3:**
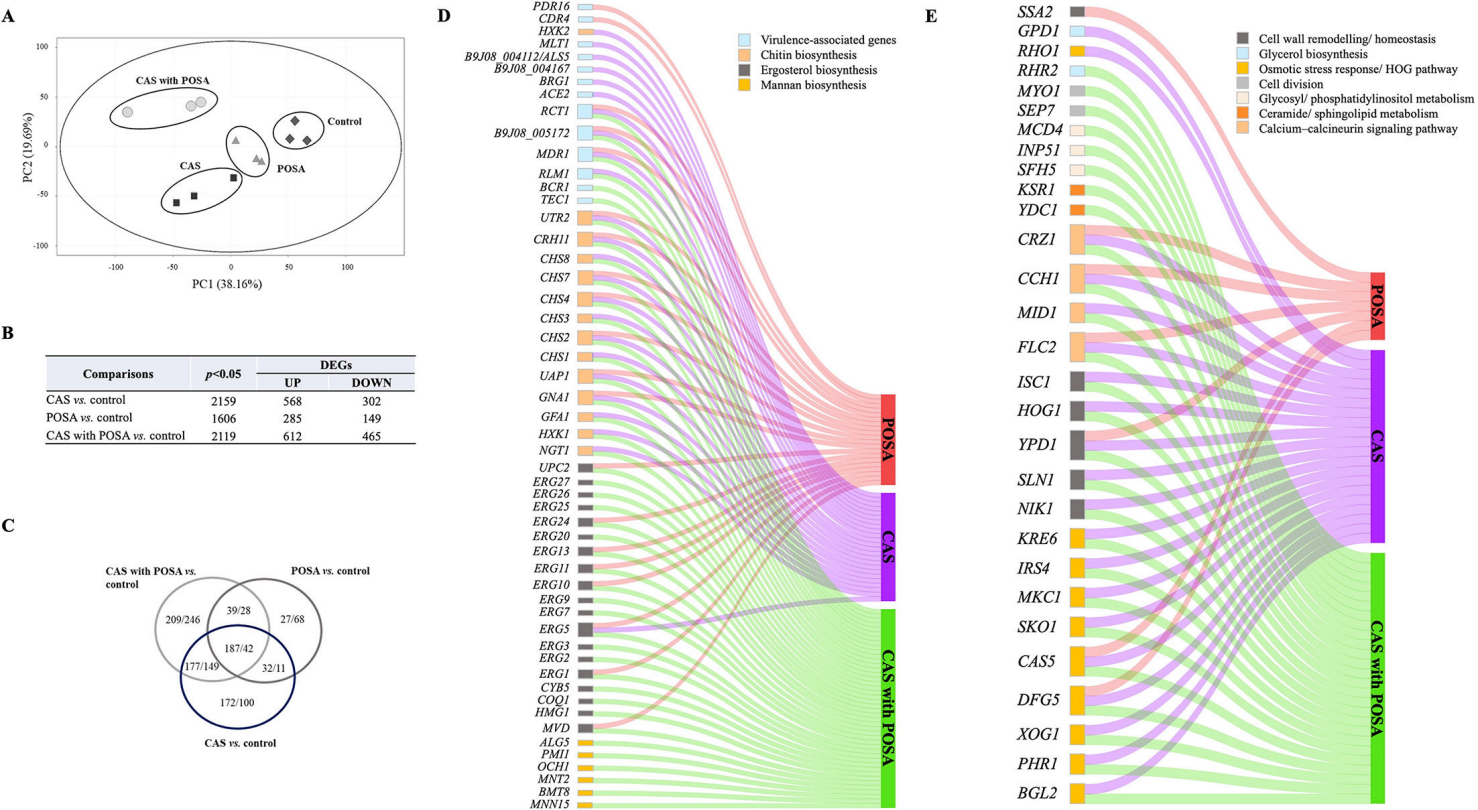
Transcriptome profiles of *Candida auris* clinical isolate. (**A**) Principal component analysis (PCA) plot of three biological replicates per caspofungin (CAS), posaconazole (POSA), and caspofungin with posaconazole (CAS with POSA) treatments. (**B**) Number of significant (*P* < 0.05) and upregulated (*P* < 0.05; FC > 1.5) and downregulated (*P* < 0.05; FC < −1.5) genes (DEGs). (**C**) A Venn diagram represents the overlap and non-overlap between upregulated/downregulated DEGs in the treated and untreated isolates. (**D and E**) The Sankey diagrams illustrate the representative genes (*P* < 0.05; FC > 1.5) within selected gene groups in the treated isolate.

To investigate transcriptional responses to antifungal treatment, total RNA-Seq was performed on *C. auris* (isolate 10) biofilms following exposure to caspofungin and/or posaconazole. One-day-old biofilms were cultured in the absence (control) or presence of sub-inhibitory concentrations of caspofungin (1 mg/L) and posaconazole (0.25 mg/L) for 24 h. Principal component analysis (PCA) was employed to assess variance and reproducibility among biological replicates and treatment groups (Fig. 3A). Replicates clustered closely within each group, indicating a high degree of consistency. In contrast, samples from antifungal-treated groups clustered distinctly from the untreated controls, reflecting specific transcriptomic changes induced by the treatments (Fig. 3A).

Overall, treatment with caspofungin and posaconazole resulted in notable transcriptomic changes in *C. auris* biofilms (Fig. 3 and Table 2; Tables S1 and S2). Specifically, 568 genes were upregulated, and 302 genes were downregulated (fold change [FC] > 1.5 or < –1.5, respectively) following caspofungin exposure, while 282 genes were upregulated and 149 genes were downregulated in response to posaconazole alone (Fig. 3B). In the case of combination, 612 genes showed increased transcription, and 465 genes were downregulated relative to the untreated control biofilms (Fig. 3B). For the 14 genes selected for RT-qPCR validation (Table S1), transcription levels measured by RT-qPCR showed a strong correlation with the RNA-Seq data, as indicated by Pearson’s correlation coefficient ranging from 0.90 to 0.92 (Table S1).

**TABLE 2:** Transcriptional behavior of selected gene groups^*a*^

Gene sets	CAS vs control	POSA vs control	CAS with POSA vs control
Cell wall organization or biogenesis (133)	Up (32 genes)	Up (16 genes)	Up (40 genes)
Chitin biosynthetic process (14)	Up (8 genes)	Up (4 genes)	Up (8 genes)
Ergosterol biosynthetic process (26)	–	Up (8 genes)	Up (19 genes)
Peroxisome (70)	Up (26 genes)	–	–
Fatty acid beta-oxidation (9)	up (9 genes)	–	–
Calcium ion transport (11)	Up (4 genes)	Up (3 genes)	Up (4 genes)
Zinc ion binding (287)	Up (36 genes)	Up (27 genes)	Up (42 genes)
Zinc ion transport (6)	Down (3 genes)	–	Down (3 genes)
Iron ion binding (47)	Up (11 genes)	Up (8 genes)	Up (13 genes)
Iron ion transport (17)	Down (3 genes)	Down (1 gene)	Down (9 genes)
Sulfite transport (2)	Up (2 genes)	Up (2 genes)	Up (2 genes)
Sulfur compound biosynthetic process (61)	–	Down (7 genes)	–
Response to osmotic stress (69)	Up (13 genes)	Up (8 genes)	Up (10 genes)
Response to oxidative stress (87)	Down (10 genes)	–	Down (15 genes)
Mitochondrial DNA repair (4)	Up (2 genes)	–	Up (2 genes)
Cell division (144)	Up (20 genes)	Down (8 genes)	

^*a*^ “Up” and “down” indicate enrichment in the upregulated and downregulated gene sets, respectively. The “–” symbol indicates no significant enrichment. The number of genes in each cluster is indicated in parentheses. The complete list of gene groups is provided in Table S2.

### Caspofungin-associated transcriptomic changes in *Candida auris* biofilm

Transcriptomic analysis demonstrated that caspofungin induced a broad stress and compensatory response in *C. auris* characterized by the upregulation of genes related to biofilm formation, cell wall remodeling, antifungal stress tolerance, osmotic adaptation, and chitin biosynthesis (Fig. 3 and Table 2; Table S2).

Caspofungin exposure also resulted in enrichment of upregulated genes involved in several key metabolic and stress-related pathways. Notably, genes associated with peroxisome function (23 genes) and fatty acid β-oxidation (*POX1-3*, *PXP2*, *ECI1*, *FAA4*, *FAA2-3*, *FAT1*, and *POT1*) were significantly upregulated. Enhanced expression was also observed in genes related to glycolysis, such as *TYE7* (transcription factor), *PFK1*, *PFK2*, *GPM1*, *CDC19*, and *HXK2*. Genes involved in sphingolipid biosynthesis (*FLC2*, *MIT1*, *FEN1*, *IPT1*, *LAG1*, and *ISC1*) were upregulated, alongside genes of the glyoxylate cycle, such as *CIT1*, *ICL1*, *MLS1*, and *MDH1–3*. In addition, caspofungin treatment induced the expression of genes related to metal ion binding, including 36 zinc ion-binding and 11 iron ion-binding genes. Genes associated with sulfite transport, such as *SSU1* and B9J08_004533, were also upregulated (Table 2; Table S2).

In contrast, genes involved in primary alcohol metabolic process (6 genes), response to oxidative stress (10 genes), antioxidant activity (7 genes), DNA replication (11 genes), and zinc ion transport (*ZRT2*, *ZRT3*, and B9J08_003341) were significantly downregulated. Notably, the expression of *SFP1* (a C2H2-type zinc finger transcription factor) and *FKS2* (encoding β-1,3-glucan synthase) was markedly reduced following caspofungin treatment (Table 2; Table S2).

### Posaconazole-associated transcriptomic effect in *Candida auris* biofilm

The posaconazole exposure alone resulted in the upregulation of several ergosterol biosynthesis genes and a subset of chitin biosynthesis-related genes. Additionally, genes related to osmotic stress response and the calcium–calcineurin signaling pathway were also enriched (Fig. 3 and Table 2; Table S2). Furthermore, 27 zinc ion-binding genes, 8 iron ion-binding genes, as well as genes involved in sulfite (*SSU1* and B9J08_004533) transport were upregulated. Genes associated with antifungal stress response, such as *PDR16* (encoding a phosphatidylinositol transfer protein) and *CDR4* (drug transport), also showed increased expression following posaconazole treatment (Fig. 3 and Table 2; Table S2).

Conversely, genes involved in primary alcohol metabolic process (three genes), fatty acid biosynthesis (five genes), fatty acid degradation (five genes), and sulfur compound biosynthesis (seven genes) were enriched in the downregulated gene set (Fig. 3 and Table 2; Table S2).

### Caspofungin with posaconazole-associated transcriptomic response in *Candida auris* biofilm

The combination of caspofungin and posaconazole induced the upregulation of a broad set of genes related to cell wall organization or biogenesis (40 genes), osmotic stress response (10 genes), the calcium-calcineurin signaling pathway, and the chitin biosynthetic process. Genes associated with biofilm development were also significantly upregulated (Fig. 3 and Table 2; Table S2). Notably, significant upregulation was observed in the case of the biofilm formation (*ZCF32*, *TEC1***,** and *BCR1,* encoding transcription factors); and for multiple genes participating in mannan biosynthesis, ceramide/sphingolipid metabolism; and glycosyl/phosphatidylinositol metabolism. Genes encoding structural and stress-related proteins, including *SEP7* and *MYO1* (septin and myosin II), were similarly upregulated (Fig. 3 and Table 2; Table S2). In addition, combination treatment resulted in the enrichment of upregulated ergosterol biosynthesis genes (19 genes), those related to zinc (42 genes) and iron (13 genes) ion binding (Fig. 3 and Table 2; Table S2). Notably, the expression of *NPR1* (nitrogen permease reactivator 1 kinase) and *HAC1* (major regulator of unfolded protein response) was markedly induced following combination treatment (Fig. 3 and Table 2; Table S2).

In contrast, caspofungin with posaconazole exposure significantly downregulated the expression of genes associated with iron ion transport (9 genes), zinc ion transport (2 genes), glycolysis (5 genes), pyruvate metabolism (22 genes), primary alcohol metabolism (7 genes), the pentose phosphate pathway (9 genes), fatty acid degradation (17 genes), and oxidative stress response (15 genes) (Table 2; Table S2). Importantly, a marked transcriptional reduction was also observed for *AHR1* (transcription factor involved in modulation of adhesion genes), *UME6* (transcription factor in biofilm dispersion), *ZCF4* and *KAR4* (transcription factors), *FKS2* (encoding β-1,3-glucan synthase), and *MCT1* (monocarboxylate transporter gene), following the combined antifungal exposure (Table 2; Table S2).

### Antifungal drugs significantly influence the intracellular metal contents of *Candida auris* biofilms

**Fig 4:**
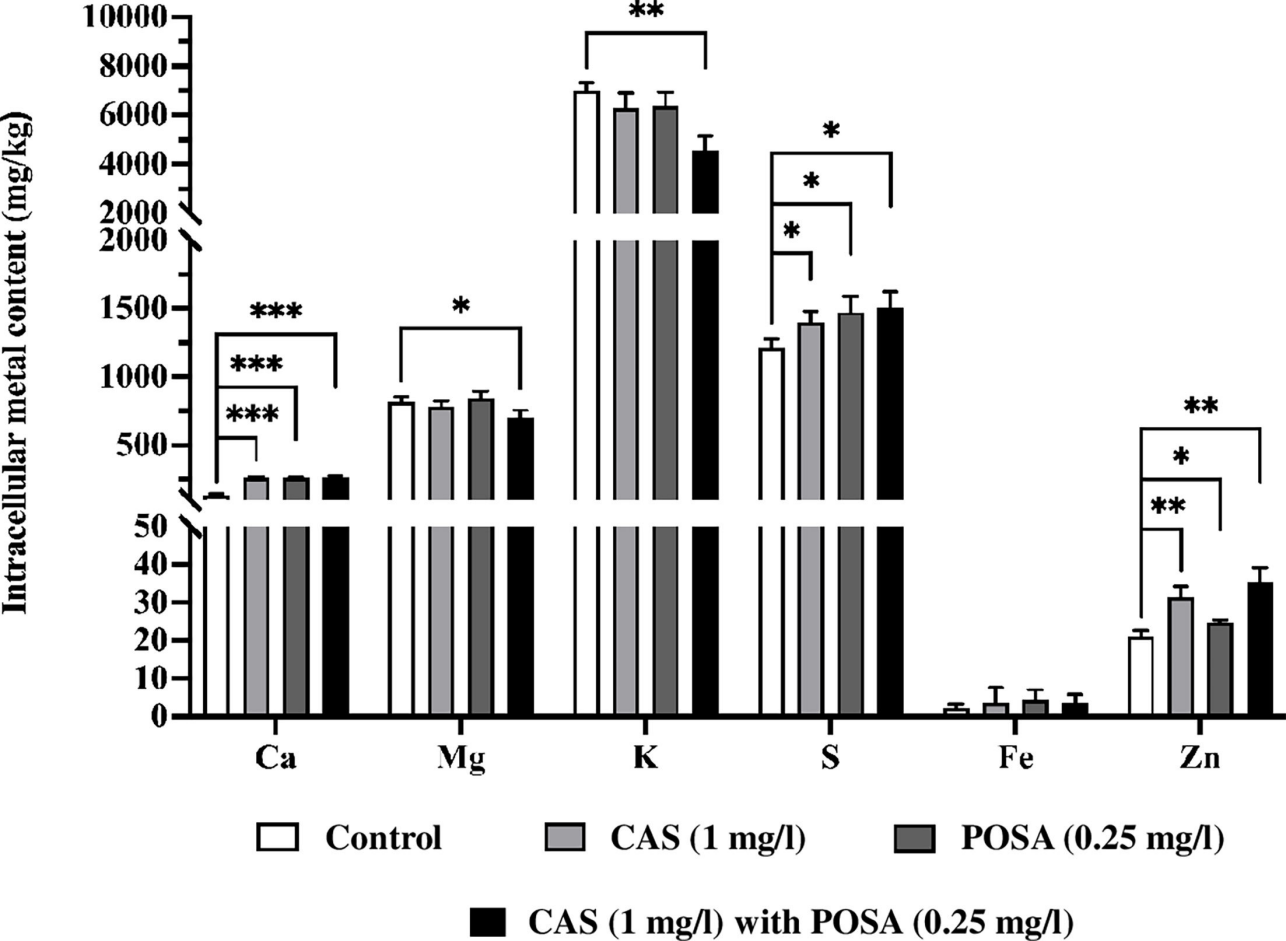
The physiological effects of caspofungin and/or posaconazole exposure on the elemental composition of *C. auris* biofilms. Metal contents were determined by ICP-OES. Data represent mean ± SEM (standard error of mean) from three independent experiments. Asterisks indicate significant differences compared to untreated controls, as determined by Student’s *t*-test: *P < 0.05, **P < 0.01, and ***P < 0.001.

Exposure to caspofungin, posaconazole, and their combination significantly altered the elemental composition of *C. auris* biofilms. Notably, calcium levels increased to 261.7  ±  11.5, 262.0  ±  5.0, and 265.8  ±  10.5  mg/kg, respectively, compared to untreated controls (133.1  ±  14.6  mg/kg). Similarly, sulfur content rose to 1,396.8  ±  82.4, 1,473.5  ±  114.5, and 1,505.2  ±  118.0  mg/kg, relative to 1,210.6  ±  64.5  mg/kg in controls. Zinc levels were also elevated, with values of 31.5  ±  2.7, 24.8  ±  0.6, and 35.5  ±  3.7  mg/kg in treated biofilms vs 21.0  ±  1.6  mg/kg in untreated samples (Fig. 4). In contrast, significant reductions in magnesium and potassium concentrations were observed in biofilms treated with the combination therapy (702.1 ± 52.5 and 4,548.9 ± 592.7 mg/kg) compared to control biofilms (816.4 ± 37.7 and 6,993.5 ± 324.9 mg/kg). Although intracellular iron levels showed a slight increase following treatment, the differences were not statistically significant compared to the untreated controls (Fig. 4).

### *In vivo* efficacy of caspofungin and posaconazole against *Candida auris*

**Fig 5:**
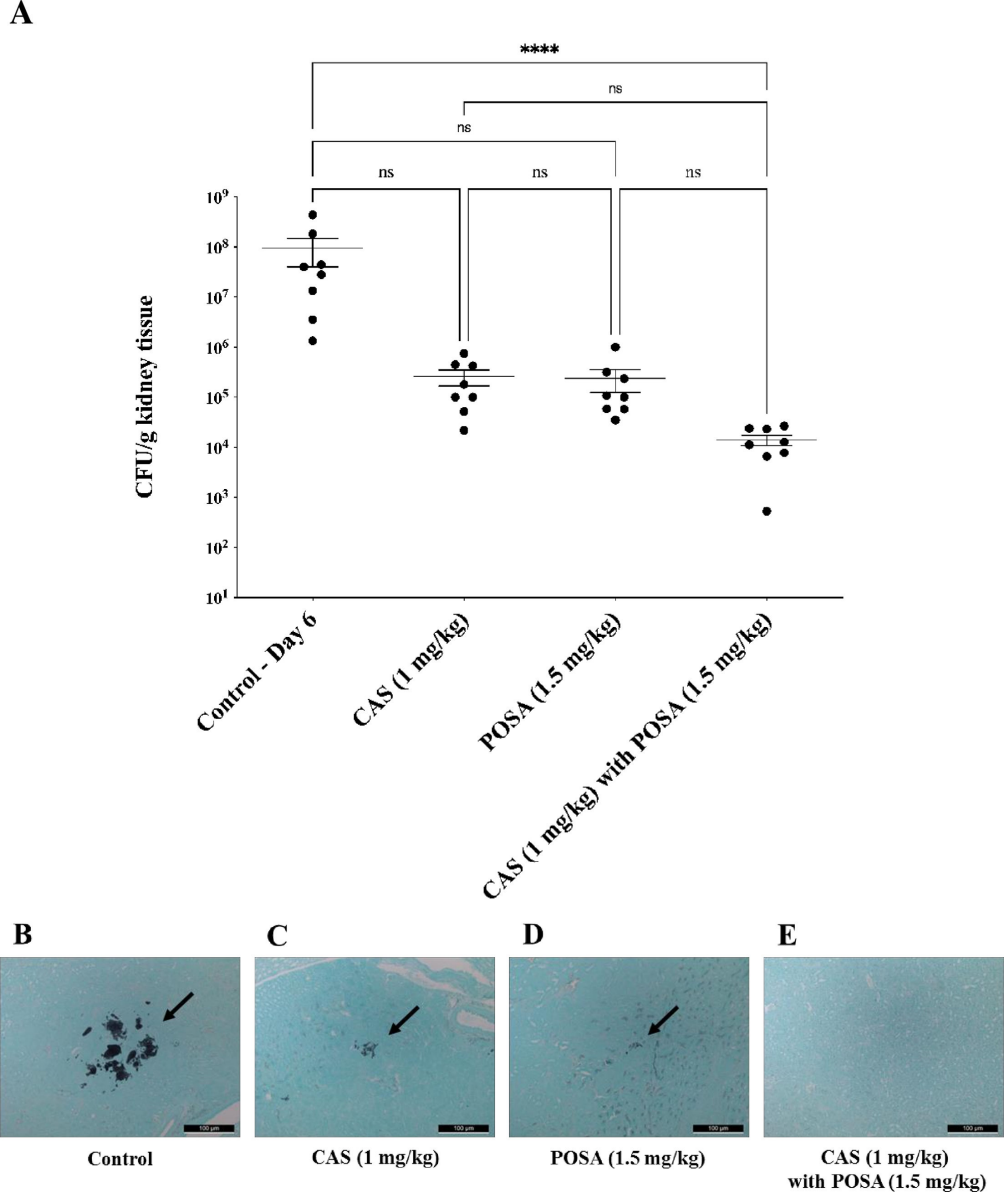
The kidney burden of *Candida auris* in a systemically infected mouse model. (**A**) The effect of 7 days of antifungal treatments on the CFU in mice infected with isolate 10. The bars represent the means ± SEM (standard error of mean) of kidney tissue burdens of BALB/c mice. Level of significant difference is indicated at *P* < 0.0001 (****); ns, not significant. (**B–E**) Histopathological examination of kidney tissues with Grocott methenamine silver staining from caspofungin (CAS) and/or posaconazole (POSA)-treated mice suffering from systemic candidiasis. Scale bars represent 100 µm. Arrows show the fungal lesions.

The *in vivo* efficacy of the treatments is presented in Fig. 5. Monotherapy with caspofungin (1 mg/kg/day) or posaconazole (1.5 mg/kg/day) resulted in a reduction in kidney fungal burden for isolate 10; however, these reductions were not statistically significant compared to the untreated control (*P*  > 0.05) (Fig. 5A). In contrast, combination therapy led to a reduction of more than 3 log_10_ CFU/g of kidney tissue, representing a statistically significant decrease compared to the control group (*P* < 0.001) (Fig. 5A). Histopathological examination corroborated these findings (Fig. 5B through E). While fungal aggregates were still present in the kidneys of animals treated with monotherapies (Fig. 5C and D), both the number and size of fungal lesions were notably reduced compared to untreated mice (Fig. 5B). Remarkably, combination therapy substantially diminished fungal infiltrates, indicating improved efficacy (Fig. 5E).

## DISCUSSION

The development of effective antifungal combinations represents a promising alternative to conventional monotherapy for the treatment of *C. auris* biofilm-associated infections. Among the various antifungal combinations investigated, the *in vitro* activity of caspofungin combined with azoles has attracted the most attention in studies involving *Candida* planktonic cells, biofilms, and animal models (24, 36, 37). To the best of our knowledge, the *in vitro* synergistic activity of posaconazole and caspofungin against *Candida* species has been explored in only a limited number of studies (19, 20, 22–24, 38). Based on previous reports, the combination of caspofungin and posaconazole demonstrated efficacy rates of 100%, 18%–85%, and 13%–50% against planktonic isolates of *C. albicans*, *C. glabrata,* and *C. auris*, respectively. In our study, this combination showed synergistic interaction in 17% of tested isolates using checkerboard microdilution, especially based on FICI determination. However, the *in vivo* effects and underlying molecular mechanisms of this combination against *C. auris* remain largely unexplored. In our study, we observed *in vitro* synergy between the caspofungin and posaconazole against *C. auris* sessile cells as determined by the checkerboard microdilution method, particularly based on FICI values. These findings were further corroborated by time–kill curve analysis. The *in vitro* synergistic interaction between antifungal agents was further evaluated *in vivo* using an immunocompromised mouse model to assess its potential correlation with therapeutic efficacy. Although monotherapy with antifungals reduced the fungal burden in the kidneys, only the combination treatment resulted in a statistically significant reduction compared to the untreated control group (*P* < 0.0001). To date, *in vivo* studies investigating combination-based therapy against *C. auris* remain limited. Specifically, Nagy et al. (2021) reported that daily administration of 1 mg/kg caspofungin combined with 20 mg/kg isavuconazole significantly improved the survival of mice infected with *C. auris* (25). More recently, Hernando-Ortiz et al. evaluated the *in vivo* efficacy of amphotericin B and echinocandin combinations, demonstrating a protective effect in *Caenorhabditis elegans* infected with *C. auris* isolates (39). Taken together, these findings suggest that the observed synergistic interactions may be further elucidated through transcriptomic analysis.

Our findings are consistent with a previous study characterizing the antifungal susceptibility and proteomic responses of *C. albicans* biofilms following caspofungin exposure (40). Notably, both *C. albicans* and *C. auris* biofilms exhibited greater resistance to caspofungin compared to their planktonic counterparts (25, 40). Furthermore, caspofungin treatment led to the downregulation of several genes related to cell wall maintenance and oxidative stress, while markedly upregulating genes involved in cell wall integrity—such as *RHO1,* a key sphingolipid-dependent regulator of cell wall integrity signaling (40–42)—as well as genes associated with metabolic pathways including glycolysis, the glyoxylate cycle, fatty acid β-oxidation, and chitin biosynthesis. These observations support the hypothesis proposed by Hoehamer et al. and Li et al. that increased energy demand is required for stress adaptation during caspofungin exposure (40, 41). In contrast, the utilization of non-sugar carbon sources—such as β-oxidation—appears to be important for *C. auris* virulence and metabolic flexibility in the presence of caspofungin, but not in response to posaconazole or the combination of both agents (43, 44).

Genes upregulated in biofilms grown in the presence of posaconazole were uniquely enriched for ergosterol biosynthesis pathways and were also associated with the cell wall chitin biosynthesis, similar to the transcriptional profile observed in caspofungin-treated biofilms. As is well established, ergosterol is essential for yeast cells, contributing to membrane fluidity, permeability, and structural integrity (44, 45). However, posaconazole induces membrane stress by disrupting sterol composition and increasing plasma membrane fluidity (46, 47). In response to such membrane stress, upregulation of ergosterol biosynthesis genes constitutes a known protective mechanism of *C. auris* biofilms against azole exposure. Moreover, elevated chitin biosynthesis and increased expression of cell wall repair proteins have also been associated with reduced susceptibility to both echinocandins and azoles, *in vitro* and *in vivo* (40, 48, 49).

Regarding additional molecular events, exposure to caspofungin in combination with posaconazole induced structural alterations affecting both the extracellular matrix and the fungal cells within the biofilm. It is well known that biofilm-embedded cells are protected by a self-produced extracellular matrix, which contributes significantly to their resistance against antifungal agents. In our study, several key regulators of matrix production—*BGL2*, *PHR1*, *XOG1*, and *RLM1*—along with transcriptional factors such as *ZCF32*, *TEC1*, and *BCR1*, were found to play important roles in the antifungal response. Previous studies have shown that antifungal agents can serve as substrates for drug transporters, and their overexpression may result in cross-resistance among structurally unrelated compounds. Interestingly, in contrast to planktonic conditions, we did not observe statistically significant overexpression of ABC transporter genes—including *CDR1*, B9J08_002451 (*CDR2*), *CDR4*, *STE6*, and *SNQ2*—with the exception of the *MDR1* gene, which was upregulated (29–31). Similar findings have been reported by Ramage et al. and Alves et al. in studies examining the response of *C. albicans* and *C. glabrata* biofilms to fluconazole (50, 51). Additionally, transcription of *FKS2*, which encodes a β-1,3-glucan synthase, was significantly downregulated in both caspofungin-treated and caspofungin–posaconazole-treated biofilms compared to the untreated control. These findings suggest that combination therapy may reduce β-1,3-glucan deposition in the biofilm matrix and cell wall, thereby impairing extracellular matrix synthesis and biofilm maturation. This ultimately leads to a reduced biofilm burden and enhanced susceptibility to antifungal agents.

The observed synergistic interaction may, in part, be attributed to the extensive osmotic stress induced by the combined disruption of membrane and cell wall integrity caused by posaconazole and caspofungin. Our study revealed transcriptional alterations in genes involved in the biosynthesis of key structural components of the cell wall and membrane—including the mannan-glucan complex, chitin, sphingolipid, and ergosterol—suggesting a compensatory response to the antifungal effects of echinocandin and posaconazole.

Moreover, our molecular findings support previous observations in *C. albicans* treated with caspofungin (42), in which interference with the interaction between phosphatidylinositol-(4,5)-bisphosphate and Rho1p at the plasma membrane—triggered by caspofungin and posaconazole—led to aberrant cytokinesis, disruption of the cell-wall integrity pathway, and mislocalization of phosphatidylinositol-(4,5)-bisphosphate, Rho GTPase (Rho1), septin (Sep7), and myosin-II (Myo1). Notably, Rho1, a GTPase that serves as a central regulator of β-1,3-glucan synthase, was strongly upregulated in biofilms but only modestly induced in planktonic cells in response to caspofungin monotherapy (42). Based on these results, we hypothesize that *RHO1* activation is a key component of the cellular response to caspofungin, both alone and in combination with posaconazole, and is consistent with the increased demand for glucan synthesis under antifungal stress.

In addition, we identified several genes as essential for the cellular response to combination treatment, including *CAS5* (a key transcriptional regulator), *HOG1* (a MAP kinase in high osmolarity glycerol pathway), *GPD1* and *RHR2* (involved in glycerol biosynthesis), *HAC1* (regulator in unfolded protein response), and *CRZ1* (an effector of calcium–calcineurin signaling pathway). Interestingly, no significant change was observed in the expression of *HSP90*, a molecular chaperone known to play a critical role in stress adaptation, antifungal resistance, and virulence in *Candida* species (40, 52).

Finally, the transcriptomic data obtained in the present study revealed that combined exposure to caspofungin and posaconazole significantly affected the expression of calcium and potassium transport-related genes, including *MID1*, *CCH1,* and *TRK1* (fold change = 1.31), as well as the intracellular levels of calcium, magnesium, and potassium in *C. auris*. These cations are known to play a critical role in the extracellular matrix of fungal biofilms, influencing their maturation, structural integrity, and resistance to antifungal drugs. Additionally, they are involved in key cellular processes, including enzyme activity regulation and osmotic homeostasis (53, 54). Previous studies have shown that magnesium deprivation inhibits the efflux activity of Cdr1 and Cdr2 transporters without affecting Mdr1 and is also associated with reduced ergosterol content (55).

Therefore, our findings provide deeper insight into the physiological and molecular mechanisms underlying the activity of a potential antifungal combination therapy. Specifically, our results highlight that the activation of multiple stress responses, protective extracellular matrix production, cell wall and membrane remodeling, maintenance of calcium homeostasis, and *MDR1*-mediated drug efflux play pivotal roles in the survival and virulence of *C. auris* biofilms during exposure to caspofungin and posaconazole—at least in isolates belonging to the South Asian clade. This knowledge may contribute to the development of safe and effective treatment strategies against recalcitrant *C. auris* biofilm-associated infections.

## Data Availability

RNA-Seq data are available in the Gene Expression Omnibus (GEO; http://www.ncbi.nlm.nih.gov/geo/) under the accession number GSE302377.

## SUPPLEMENTAL MATERIAL

Table S1 spectrum.02686-25-s0001.xlsx Overview on *Candida auris* isolates and RT-qPCR assays.

Table S2 spectrum.02686-25-s0002.xlsx Results of gene set enrichment analyses.

ASM does not own the copyrights to Supplemental Material that may be linked to, or accessed through, an article. The authors have granted ASM a non-exclusive, world-wide license to publish the Supplemental Material files. Please contact the corresponding author directly for reuse.
